# General Care of the Mouth for Patients under Twenty Years of Age

**Published:** 1880-10

**Authors:** L. C. Taylor

**Affiliations:** Hartford, Conn.


### ARTICLE II.

### *General Care of the Mouth for Patients under Twenty Years of Age.*

BY DR L. C. TAYLOR, OF HARTFORD, CONN.

When I first received the invitation from your committee to read a paper before this assembly, I was inclined to reply in the negative, but the more I thought of it the more I thought this subject ought to be pressed in the Society, and the experience of elder members drawn out by discussion.

When I first commenced the practic of dentistry, I had very little idea of the causes of decay, and much less of the general diseases of the mouth. I had often heard them spoken of, but gained no knowledge more than that decay was present in nearly every mouth, and the teeth ought to be filled. I was then very sanguine that, if they would only have me fill their teeth, they were safe for the rest of their natural life. To be sure I was honest in my belief, and in all cases tried to perform operations faithfully.

I very soon found that operations performed in advanced life were far more durable than those performed for younger people. I filled teeth, every cavity I could find, and, like many others, recommended the preservation of all six-year molars if they were not so far gone that I could not fill them; and, even then, was so strong in my belief of saving teeth, that I would fill one or three, when the others were lost from an imperative necessity. The age of the patient made very little difference. God had designed that we have thirty-two teeth, and surely the All-Wise had made no mistake. If we violate nature's laws, and fail to develope to the full stature as designed, the fault is not His, but ours. Every tooth that showed signs of decay presented a sufficient reason for a filling.

Of course, the question very naturally came to my mind, What was the cause of such immediate failures in young patients? I am convinced that continued disease of the soft parts has much to do with the tendency to decay; in fact, a very large percentage of the decays now existing in the mouths of the human family are directly traceable to the fevered condition of the soft parts.

If it were not so, why should we find the mouths of so many of our patients nearly or quite healthy on one side, and on the other from ten to fifteen cavities. If we will study such cases, we will very soon find our text; yea, we shall find a whole chapter.

We will invariably find, where a case is like the one referred to, that the patient has lost, while young, a tooth on the side where the greatest degree of health exists. Gentlemen, I am a believer in the thinning-out process for very many of our younger patients, especially those under sixteen. We are told by some eminent authority that the thinning-out process may do for the gardener when he is raising his carrots and beets, but in no instance is it advisable in the general care of the mouth.

I have been asked if I believed in thinning out the mouths of all patients. I answer, no. If nothing need be done, do nothing; but it is my firm belief that twenty-eight good teeth are far better than thirty-two poor, patched up ones. As I have alluded to the six-year molars, the impression may be drawn that I adopt the extraction of these teeth, without regard to their quality, or the age of the patient. Generally, the necessity for thinning out may be anticipated before twelve-year molars are erupted; but never until the second bicuspids have all made their appearance, as they may turn a quarter around if the lateral force is relieved so as to allow the cusps of the new teeth to turn on the roots of the deciduous ones.

When the patient has been neglected until the teeth have all made their appearance (wisdom teeth excepted,) the quality of the various teeth should determine what to extract, extraction always being done systematically, relieving both sides of the mouth so as not to drive the mesial line to the right or left.

If the dentist has the good of his patient at heart, he had better thin out the teeth for one-half of his city patients, and one-third of those from the country.

Our city children are not usually as strong as those reared out of the city. The mental strain upon the system is not as great on the scholar who is in school only twenty-four weeks in the year as on one who is in forty. Our country children, who are reared in the open air, directly under the beautiful sky of heaven, with the sun shining upon them, will be far more vigorous, both physically and mentally, than if enclosed in one of our crowded city schools. Circumstances ought to be considered before we form our opinions.

The ancestry of each and every patient, where there is a shadow of a doubt as to the best course to pursue, should be considered, which may aid to a great extent in determining how far nature is going to help us. If the father be large in his osseous stature, and the mother the reverse, with a somewhat V-shaped arch, we may expect but little, especially if such is the general make up of the preceding generations; but if the reverse be presented, then we may look for some improvement as the patient advances in life.

Irregularities should always be considered, for no patient wants to develope into manhood or womanhood with distorted and unsightly features. Cases are very rare where there is any necessity for badly-deformed features, unless it arises from some other cause than the teeth. Very little assistance need be rendered the masses if that little be given at just the right time. In order to do this, it is necessary that the dentist be well versed in the development of the dental organs. He should also be well acquainted with the law of forces, for when force is applied to the teeth it is sure to have its effect, and if the force be applied in the wrong direction, the results are sure to be bad. Whenever the teeth are badly irregular, we are very apt to consider them badly crowded, and a necessity for relief is very apparent to almost every one.

We have briefly referred to irregularities as one of the causes for extraction. I wish now to make allusion to some of the worst crowded mouths we have to deal with. The teeth in these mouths are not irregular, but even, and often beautiful.

The amount of force that is applied in the developement of the twelve-year molars and wisdom teeth is enormous, and is very likely to pass unnoticed by very many of our so-called first-class practitioners. When a dentist claims that the teeth have a right to be crowded, as long as they are regular, I fear he is overlooking some very important feature in the case, which, if allowed to develope, will be conducive of a great amount of suffering. If the gums are allowed to remain in such a fevered condition as they most likely will assume from the crowded state of the teeth, tartar will be very prevalent; also, the green coating, called by some of our best authorities fungus. Some consider the coating an indication of acidity of the stomach, but it is more probable that it is a fungus, induced by a slightly unhealthy secretion emitting from the gums. That such is a fact is proved by faithfully removing the coating, and restoring the gums to perfect health by giving the teeth relief. Whenever the coating is removed, and the teeth all retained, we very soon find it gradually returning in mouths of patients who use great care.

There is not an intelligent man in this audience who will not admit that tartar is one of the worst enemies we have to deal with. Of late years we have heard a great deal about diseased gums. Without doubt, a very large percentage of diseased gums is traceable to tartar. Tartar usually gets its first hold in quite young patients, owing to the fever produced in the surrounding parts by the eruption of those teeth which make their appearance later in life.

During the changes of the whole dentition, it is of great importance that we insist upon thorough cleanliness. No patient under twenty ought to allow more than six months, or one year at the longest, to pass without visiting his dentist and having his teeth thoroughly cleaned. I do not mean five or ten minutes with the stick and pumice is sufficient, or even less time with the rubber disk, which is so commonly used by our modern machine dentists. I mean that we should use such instruments as will best remove all accumulations of tartar, and break up the coating so generally found. The coating should be removed to the extreme edge, quite often extending under the gums a short distance. The best evidence that we have thoroughly accomplished our purpose will be the perfect health of the gums, which will assume a pink color, instead of the angry red, on the margin.

If we see striated streaks in the gums, either inside or outside, we may know that tartar is present in the form of a little nodule, which can be dislodged very easily. If allowed to remain, it will induce other deposits, until inflammation absorbs away the principal portion of the process, and the teeth become loosened, as we so often find in the mouths of patients in middle life and advanced age.

Modern dentistry aims to ward off these pending troubles. To do this, we must be thoroughly conversant with nature, and be able to diagnose the incipient stages, as that is the time we can do the greatest amount of good.

One eminent English author has been recorded as saying that diseased gums were seldom found in the mouths of patients under thirty years of age. We should consider it very absurd should any one make the remark that cavities were seldom present where the patient was under twenty, yet it is no more absurd than the remark referred to.

The slightest mechanical or chemical irritation producing any change from the normal color may be considered an indication of diseased gums, and should receive such treatment as to remove the cause, and the effects will take care of themselves. There is not an intelligent mind in this community who would not scout the idea of a surgeon's prescribing iodine, tincture of myrrh, or any other therapeutic remedy, for a sore on the hand, caused simply by the lodgment of a sliver. Many, who are considered good practitioners, are doing precisely the same thing every day by making this kind of application to the gums, when nothing is needed more than in the case of the surgeon. Remove the sliver, and the wound very soon heals. Remove the tartar, and nature will perfect a cure, unless there has been a wasting of the osseous parts which nature will be able to restore.

The point I wish to make here is the importance of diagnosing all diseases in their incipient stages, and treating them then. The importance of looking after the health of the mouth is equally as great as filling cavities. It is said that "an ounce of prevention is worth a pound of cure." The same is true in this case ; if we maintain perfect health, there will be little or no decay.

My own observation has proved that, in some localities where exostosis is prevalent, crowded teeth and inflamed tissues are especially favorable to this much-dreaded disease. When an osseous tumor is well formed, there seems to be no cure except in extracting the offending member. Neuralgia of the face and head are very often the fruits of such disease. Exostosis is a disease not generally recognized by the profession until the tooth has been removed, and the disease exposed to the eye. I was once considerably surprised when an elderly practitioner informed me that there was well-defined exostosis in the mouth of a certain patient.

He then called attention to the peculiar red margin of the gums, at the same time remarking that the majority of dentists would say that diseased gum, in its early stages, was caused by tartar. After questioning the patient, we found she was at times an intense sufferer from neuralgia, which was one of the proofs of a correct prognosis. The four sec-second bicuspids were removed, and all of them showed well-defined exostosis. Since the removal of these teeth, the patient informs me she has never been troubled with neuralgia. It is not at all reasonable to suppose that there are no other teeth in the mouth referred to that are not affected. The relief of the lateral forces has effected a cure for the present, but that it never will trouble her again is more than any one can affirm with any degree of certainty. If that mouth had been thinned out before a sufficient amount of inflamation had been produced to invite hypertrophy of the cementum, there is no doubt the cure would have been permanent, as the disease would never have developed beyond a slight inflammation.

To be successful in conducting our young patients over what is called the critical age, requires strict integrity and a well-grounded knowledge of what will be the results of general symptoms. We are too often inclined to delay taking decisive measures until the time has passed for doing the greatest amount of good to the patient. Sometimes it is very difficult to determine just what we had better do; at other times, perhaps, we are influenced by the reluctance of the little patient, who shrinks from submitting to that which will do the greatest amount of good. If we first gain the confidence of our young patients, and then be very careful never to deceive them in the least degree, we shall soon find them to be far better patients than those in more advanced life. A thorough explanation of the reasons why we do this and that, will be a source of education to the little one, that will mature with the child, and inspire a confidence that will retain our patient's future patronage.—*Trans. Conn. Valley D. S.*

